# Molecular Characterization of Antimicrobial Resistance and Virulence Genotypes in *Staphylococcus aureus* Isolates: Investigation of *mecA*, *pvl*, and *agr* Groups

**DOI:** 10.3390/pathogens15090957

**Published:** 2026-09-08

**Authors:** Pınar Etiz, Filiz Kibar, Hülya Binokay

**Affiliations:** 1 Abdi Sütcü Vocational School of Health Services, Cukurova University, 01330 Adana, Türkiye; 2 Balcalı Hospital Health Application and Research Center, Central Laboratory, Cukurova University, 01330 Adana, Türkiye; fkibar@gmail.com; 3 Department of Medical Microbiology, Faculty of Medicine, Cukurova University, 01330 Adana, Türkiye; 4 Department of Biostatistics, Faculty of Medicine, Cukurova University, 01330 Adana, Türkiye; hulyabinokay@gmail.com

**Keywords:** *agr* groups, antimicrobial resistance, *pvl*, *Staphylococcus aureus*, *mecA*

## Abstract

Background: This study aimed to characterize the distribution of *mecA*, *pvl* carriage, and *agr* groups, among clinical *Staphylococcus aureus* (*S. aureus*) isolates and to correlate these findings with comprehensive antimicrobial susceptibility profiles. Methods: *S. aureus* isolates—obtained from various clinical samples between 1 June 2025 and 30 March 2026 at a tertiary hospital in southern Turkey—were identified using the MALDI-TOF VITEK MS system. Antimicrobial susceptibility testing was performed using the VITEK 2 system in accordance with EUCAST standards. Target-specific Real-Time PCR was used for molecular detection of resistance and virulence genes (*nuc*, *mecA*, *pvl*, and *agr* loci (I–IV)). Results: Among the 100 isolates included, *S. aureus* identification was confirmed via real-time PCR targeting the *nuc*. The real-time PCR framework demonstrated a perfect correlation between *mecA* genotypes and phenotypic resistance profiles. The *pvl* significantly clustered with the *agr* group *II*; *pvl*-positive strains showed high resistance to erythromycin, fusidic acid, and mupirocin, while clindamycin resistance was limited to *pvl*-negative strains. Conclusions: These findings characterise the local epidemiological distribution of *pvl*, *agr* groups and resistance profiles in *S. aureus* and confirm the independent association between *pvl* carriage and the MRSA phenotype. These single-centre data highlight the need for future multicentre studies incorporating sequence-based typing, whilst providing valuable information for institutional surveillance and antibiotic management.

## 1. Introduction

*S. aureus* is a Gram-positive pathogenic bacterium that can cause serious clinical conditions, including bacteremia, endocarditis, osteomyelitis, and skin and soft tissue infections. The virulence of *S. aureus* is determined by various surface components and extracellular factors that are regulated by the global accessory gene regulator (*agr*) and exert their effects through tissue-damaging toxins such as Panton–Valentine leukocidin (*pvl*). Furthermore, methicillin-resistant *S. aureus* (MRSA) strains arising from the horizontal transfer of the *mecA*, which encodes the low-affinity penicillin-binding protein PBP2a, are one of the biggest challenges in treating this bacterium [1,2,3].

Antimicrobial resistance (AMR) is considered by the World Health Organization (WHO) and international authorities as one of the most significant public health threats globally [4]. Within this burden of resistance, MRSA is a critical pathogen that directly increases mortality and the disability-adjusted life years (DALYs) in both hospital and community-acquired infections worldwide [4,5,6]. Epidemiological data show that *S. aureus* is one of the leading causes of mortality from bacterial infections worldwide; the number of deaths directly attributed to MRSA rose from 57,200 in 1990 to 130,000 in 2021, while overall pathogen-associated mortality increased from 261,000 to 550,000 cases [4,7].

Global epidemiological data networks reveal that the prevalence of the *mecA* determinant exhibits temporal and regional fluctuations across various host populations. However, the clinical impact of methicillin-resistant strains is not defined solely by their resistance profiles, but is also closely related to specific genetic combinations. This variability in geographic distribution highlights the need to assess how resistance genes interact with different virulence markers in order to fully understand the evolutionary success of pathogenic strains [3,8,9,10].

Among the various Staphylococcal virulence factors, *pvl* stands out, a critical cytotoxin encoded by bacteriophages that triggers leukocyte destruction and tissue necrosis, predisposing hosts to deep skin and soft tissue infections [1]. Although the global prevalence of *pvl*-producing strains has shown an upward trend recently, it varies significantly across different geographic regions, ranging from 12.8% in China and 30% in Germany to 45.3% in Japan and 97% in the United States [11,12]. In vitro assessments via quantitative real-time PCR (qRT-PCR) show that some community-isolated *S. aureus* strains can exhibit extremely high levels of *pvl* expression, further highlighting the active emergence of pathogenic clones in local environments [12].

The regulation of these virulence factors is linked to the agr locus, a global signalling system that plays a role in the transition from colonisation to invasive infection [1,13]. To date, four distinct polymorphic variants (*agrI–IV*) have been identified [14]. Scientific data reveal that the distribution of these specific *agr* groups varies significantly not only between methicillin-resistant (MRSA) and methicillin-susceptible (MSSA) isolates but also in relation to the *pvl* positivity of the strains. Therefore, investigating the relationships among *agr* genotypes, methicillin resistance status, and *pvl* carriage is of critical importance for understanding the molecular epidemiological characteristics and virulence profiles of clinical *S. aureus* strains [1,10].

In light of the complex interplay between staphylococcal pathogenesis and antimicrobial resistance, a comprehensive mapping of the regional genetic landscape is of paramount epidemiological importance. Although global data reveal geographical diversity among staphylococcal lineages, comprehensive regional data clearly linking phenotypic resistance profiles to underlying viruotypes remain limited. Indeed, a recent laboratory-based study conducted in Turkey reported changing dynamics of methicillin resistance and found that the *pvl* gene had a relatively low prevalence (2%) amongst clinical isolates [15]. To the best of our knowledge, no comprehensive study has yet been documented in detail in our country that simultaneously examines the *mecA*, *pvl*, and *agr* polymorphic groups by directly correlating them with comprehensive antimicrobial susceptibility profiles. This laboratory-based study aims to fill this epidemiological gap by providing a comprehensive characterisation of the resistance and virulence profiles of *S. aureus* isolates obtained from routine clinical diagnostic samples. In particular, we sought to determine the prevalence of key staphylococcal determinants, including the species-specific *nuc*, the cassette-derived *mecA*, *pvl*; at the same time, we mapped the associated *agr* polymorphic groups (I–IV). More importantly, this study focused on conducting a comprehensive cross-analysis of these genotypic profiles with in vitro antimicrobial susceptibility patterns, thereby providing insight into local epidemiological characteristics and the concurrent carriage of resistance and virulence determinants. Consequently, the epidemiological data obtained aim to provide information for improving institutional empirical treatment strategies and strengthening regional infection control protocols.

## 2. Materials and Methods

### 2.1. Study Design and Bacterial Isolates

This prospective laboratory-based study was conducted at the Cukurova University Balcalı Hospital Health Practice and Research Center, Central Laboratory. A total of 100 non-duplicate *S. aureus* strains, isolated from various clinical specimens obtained from both outpatients and hospitalized patients in different wards and intensive care units, between 1 June 2025, and 30 March 2026, were included in the study.

### 2.2. Ethical Approval

The study was approved by the Non-Interventional Clinical Research Ethics Committee of Cukurova University Faculty of Medicine (date: 3 April 2026, and decision no: 48) and conducted according to the principles of the “Declaration of Helsinki.”

### 2.3. Inclusion and Exclusion Criteria

A total of 100 non-duplicate clinical *S. aureus* isolates recovered from 100 individual patients were included in the study. Isolates identified as *S. aureus* according to standard phenotypic and molecular criteria were evaluated. Only the first confirmed clinical isolate per patient was included in the study cohort. Duplicate, repeat, or sequential isolates from the same patient and isolates recovered from routine screening swabs (e.g., nasal or rectal surveillance), polymicrobial cultures without pure growth, and isolates that lost viability upon subculturing were systematically excluded prior to all phenotypic and molecular analyses.

### 2.4. Storage and Preparation of Isolates

All confirmed *S. aureus* isolates were inoculated into commercial bead storage systems (Microbank™, Pro-Lab Diagnostics, Richmond Hill, ON, Canada) and maintained at −20 °C until the time of molecular characterization. Before the extraction of genomic DNA and phenotypic susceptibility testing, the isolates were revived by subculturing on 5% sheep blood agar and incubated at 37 °C for 18–24 h.

### 2.5. Bacterial Identification and Molecular Confirmation

Identification of *S. aureus* was performed using conventional methods (catalase, Gram stain, coagulase) and the MALDI-TOF VITEK MS system (bioMérieux, Lyon, France). Additionally, to ensure the highest level of diagnostic accuracy, molecular confirmation of all *S. aureus* isolates was subsequently conducted via Real-time PCR by targeting the species-specific *nuc* (thermostable nuclease) locus. The sequences of the specific primers for the *nuc* amplification are detailed in Table 1.

### 2.6. Antimicrobial Susceptibility Testing and Phenotypic Identification of MRSA and MSSA

Antimicrobial susceptibility profiles of the *S. aureus* isolates were determined using the VITEK 2 automated System (bioMerieux, Marcy-l’Étoile, France) software version 9.04.4, according to the manufacturer’s instructions.

The differentiation between Methicillin-Resistant *S. aureus* (MRSA) and Methicillin-Susceptible *S. aureus* (MSSA) was primarily conducted through the automated Cefoxitin Screen test integrated into the VITEK® 2 system. The system measures the growth kinetics in the presence of cefoxitin; isolates showing growth above the resistance threshold were phenotypically classified as MRSA, while those showing inhibition were categorized as MSSA. These phenotypic results served as the preliminary classification before molecular verification of *mecA* carriage.

Phenotypic antimicrobial susceptibility testing was performed using the staphylococci-specific VITEK 2 AST-P664 test cards, which included the following agents:

- Beta-lactams: Penicillin (PEN), Oxacillin (OX), and Cefoxitin (screen)
- Fluoroquinolones: Levofloxacin (LEV)
- Macrolides and Lincosamides: Erythromycin (ERY) and Clindamycin (including inducible clindamycin resistance detection)
- Fusidanes: Fusidic acid (FUS)
- Glycopeptides and Lipopeptides: Vancomycin (VAN), Teicoplanin (TEC), and Daptomycin (DAP).
- Tetracyclines and Glycylcyclines: Tetracycline (TET), Tigecycline (TGC)
- Oxazolidinones: Linezolid (LZD)
- Nitrofuran: Nitrofurantoin
- Sulfonamides: Trimethoprim-Sulfamethoxazole (TMP/SXT)
- Other Agents: Mupirocin

Minimum inhibitory concentrations (MICs) were determined for each agent. Results were categorized as susceptible (S) or resistant (R) according to European Committee on Antimicrobial Susceptibility Testing (EUCAST) clinical breakpoints [16].

### 2.7. Quality Control

To ensure the accuracy, reliability, and reproducibility the following reference strains were utilized as quality control: *S. aureus* ATCC® 29213 (as the MSSA control strain), *S. aureus* ATCC® 43300 (as the MRSA control strain).

The nucleotide sequence of the designed primer for *mecA*, *nuc*, *pvl* and *agr* groups of *S. aureus* were verified r using the Basic Local Alignment Search Tool (BLAST, blastn suite, version 2.14.0; National Center for Biotechnology Information, Bethesda, MD, USA; available at https://blast.ncbi.nlm.nih.gov/Blast.cgi, accessed on 6 September 2026) against the GenBank® databas (Table 1) [17].

### 2.8. Genomic DNA Extraction

Genomic DNA isolation from clinical *S. aureus* isolates was performed using the PureLink™ Genomic DNA Mini Kit (Cat. No. K1820-00; Invitrogen, USA) in accordance with the manufacturer’s protocol for Gram-positive bacteria. The resultant template DNA was stored at −20 °C for PCR analysis.

Strict precautions were taken in the laboratory to prevent cross-contamination between samples and contamination caused by personnel. These precautions included the use of pre-aliquoted reagents, special gloves, and aerosol-proof filter tips. Additionally, the preparation of the master mix and post-amplification analyses were performed in separate, designated areas using separate pipette sets. To monitor for potential contamination events, nuclease-free water was used as a negative control in every PCR run.

### 2.9. Molecular Detection of Resistance and Virulence Genotypes via Real-Time PCR

Real-time PCR amplification for the qualitative detection (binary presence/absence determination) of resistance and virulence determinants (*mecA*, *pvl* and *agr* groups I–IV) was performed using a SYBR Green-based master mix (BlasTaq™ 2X PCR MasterMix; Cat. No. G891/G892; Applied Biological Materials [abm] Inc., Richmond, Canada) on a Roche LightCycler® 480 Real-Time PCR System (Roche Diagnostics, Mannheim, Germany). Each 20 µL reaction mixture was assembled on ice, containing 10 µL of 2X PCR MasterMix, 0.5 µL each of forward and reverse primers (10 µM), template genomic DNA, and nuclease-free water. The thermocycling conditions consisted of an initial enzyme activation step at 95 °C for 3 min, followed by 40 cycles of denaturation at 95 °C for 15 s, and annealing/extension at 60 °C for 1 min, and a final melting curve analysis. Because the validated primer pairs were adapted from conventional PCR protocols yielding amplicons ranging from 279 to 588 bp, this combined annealing/extension step was selected to facilitate efficient amplification across the target sequences.

The agr locus comprises two divergent transcripts, RNAII and RNAIII, which control the accessory gene regulator quorum-sensing network. RNAII encodes the *agrBDCA* operon, in which *agrD* produces the autoinducing peptide (AIP) precursor, *agrB* facilitates AIP processing and export, *agrC* acts as the transmembrane sensor kinase, and *agrA* serves as the response regulator. Polymorphisms within the hypervariable *agrB*, *agrD*, and *agrC* sequences define the four distinct specificity groups (*agr* groups I–IV) [1].

For the determination of the staphylococcal *agr* groups (I–IV), individual singleplex PCR reactions were set up for each group under the same thermocycling conditions. To achieve group-specific amplification without requiring full-length sequencing, a universal forward primer (adapted from the Pan-1 primer described by Jarraud et al., 2002) [18] was paired with distinct, group-specific reverse primers for each *agr* locus. The sequences of all specific primers used for resistance/virulence gene screening and *agr* genotyping are detailed in Table 1.

**Table 1:** Primers used for real-time PCR.

Target Gene/Locus	Primer	Primer Sequence (5′–3′)	Amplicon Size Bp	Ref.
*mecA*	*mecA* fw *mecA* rev	AAAATCGATGGTAAAGGTTGGC AGTTCTGGAGTACCGGATTTGC	533	[3]
*nuc*	*nuc* fw *nuc* rev	GCGATTGATGGTGATACGGTT AGCCAAGCCTTGACGAACTAAAGC	279	[3]
*pvl*	*pvl* fw *pvl* rev	ATCATTAGGTAAAATGTCTGGACATGATCCA GCATCAAGTGTATTGGATAGCAAAAGC	433	[1]
*agrI*	*agrI* fw *agrI* rev	ATGCACATGGTGCACATGC GTCACAAGTACTATAAGCTGCGAT	440	[19]
*agrII*	*agrII* fw *agrII* rev	ATGCACATGGTGCACATGC GTATTACTAATTGAAAAGTGCCATAGC	572	[19]
*agrIII*	*agrIII* fw *agrIII* rev	ATGCACATGGTGCACATGC CTGTTGAAAAAGTCAACTAAAAGCTC	406	[19]
*agrIV*	*agrIV* fw *agrIV* rev	ATGCACATGGTGCACATGC CGATAATGCCGTAATACCCG	588	[19]

Quality control and analytical specificity were monitored systematically throughout the assay runs: Standard reference control strains, including *S. aureus* ATCC 43300 (*mecA*-positive/MRSA control) and *S. aureus* ATCC 25923 (*mecA*-negative/MSSA control), together with no-template controls (NTCs), were included in each PCR run. Following amplification, dissociation (melting curve) analysis was performed over a temperature range of 60 °C to 95 °C. Target positivity was defined by the simultaneous presence of an exponential amplification curve with a cycle threshold (Ct) value < 35 and a sharp, distinct dissociation peak consistent with the expected amplicon-specific melting profile. Samples showing no amplification, Ct values ≥ 35, or abnormal, broad, or non-specific melting profiles were interpreted as negative. Screening was conducted using single reactions; however, isolates showing ambiguous amplification profiles, borderline Ct values, or uncertain melting peaks were systematically re-tested in duplicate to confirm the final binary result. Representative melting peak profiles for target genes (*nuc*, *mecA*, *pvl*) and *agr* groups (I–IV) are provided in the Supplementary Materials (Figures S1–S7).

### 2.10. Statistical Analysis

Data analysis was performed using IBM SPSS Statistics for Windows, Version 20.0 (IBM Corp., Armonk, NY, USA). Categorical variables were summarized as frequencies and percentages, while continuous variables were expressed as mean, standard deviation (or median and minimum–maximum range, where appropriate). Group comparisons for categorical data were evaluated using the Chi-square test. For continuous variables with non-normal distribution, differences between two groups were analyzed using the Mann–Whitney U test. To identify independent factors associated with the MRSA phenotype while controlling for demographic confounders (age and sex) and minimizing small-sample bias, a multivariable Firth’s penalized logistic regression analysis was performed. Adjusted odds ratios (aORs) and their corresponding 95% profile-likelihood confidence intervals (CIs) were calculated. Model diagnostics were evaluated using standardized Pearson and deviance residuals, leverage values obtained from the diagonal elements of the hat matrix, and generalized Cook’s distances. Absolute standardized residuals greater than 2 were considered potentially unusual, whereas values greater than 3 were considered extreme. High leverage was screened using a threshold of 2p/n, where p represented the number of estimated model parameters, including the intercept. Cook’s distances greater than 4/n were examined as potentially influential, and values greater than 1 were considered strongly influential. A case-deletion analysis was conducted to determine whether the estimated association for *pvl* carriage was dependent on any single observation. Multicollinearity was assessed using variance inflation factors. Overall model performance was evaluated using the Hosmer–Lemeshow test, calibration intercept and slope, Brier score, AUC, and bootstrap optimism correction. For all statistical tests, *p*-value < 0.05 was considered statistically significant.

## 3. Results

### 3.1. Baseline Distribution of the S. aureus Isolates

A total of 100 *S. aureus* isolates were recovered from various clinical specimens of the patients and stratified into methicillin-resistant *S. aureus* (MRSA, *n* = 55) and methicillin-susceptible *S. aureus* (MSSA, *n* = 45) cohorts (Table 2). The median age (38 vs. 41 years; *p* = 0.623) and sex distribution (*p* = 0.340) were comparable between the two groups. Similarly, no significant variance was observed regarding specimen types (*p* = 0.647), with wound specimens (52.7% vs. 51.1%) and blood cultures (27.3% vs. 24.4%) being the predominant sources.

Conversely, a statistically significant difference was identified in patient settings (*p* = 0.044). Outpatients exhibited a higher rate within the MRSA cohort than the MSSA cohort (56.4% vs. 37.8%), whereas inpatient wards showed a higher density of MSSA isolates (55.6% vs. 30.9%). ICU admissions accounted for 12.7% of MRSA and 6.7% of MSSA cases.

### 3.2. Phenotypic Antimicrobial Susceptibility Profiles

Antimicrobial susceptibility profiles stratified by methicillin resistance status are detailed in Table 3. After correction for multiple comparisons using the Benjamini–Hochberg false discovery rate procedure, statistically significant differences in antimicrobial resistance remained between the MRSA (*n* = 55) and MSSA (*n* = 45) groups. Penicillin resistance was significantly more frequent among MRSA isolates than among MSSA isolates (100.0% vs. 75.6%; FDR-adjusted *p* < 0.001). Similarly, MRSA isolates exhibited higher resistance rates to erythromycin (61.8% vs. 11.1%; FDR-adjusted *p* < 0.001) and fusidic acid (41.8% vs. 13.3%; FDR-adjusted *p* = 0.004).

Although clindamycin resistance was more frequent among MRSA isolates than among MSSA isolates (25.5% vs. 8.9%), the difference did not remain statistically significant after FDR correction (adjusted *p* = 0.064). Inducible clindamycin resistance (21.8% vs. 8.9%; adjusted *p* = 0.119) and mupirocin resistance (23.6% vs. 8.9%; adjusted *p* = 0.087) were also numerically more frequent among MRSA isolates, but these differences were not statistically significant. No significant differences were observed between the groups in resistance to daptomycin, levofloxacin, tetracycline, or trimethoprim–sulfamethoxazole (TMP-SXT) (all FDR-adjusted *p* > 0.05).

All isolates in both groups were susceptible to linezolid, nitrofurantoin, teicoplanin, tigecycline, and vancomycin; therefore, statistical comparisons were not performed for these agents. As expected, cefoxitin-screen positivity and oxacillin resistance, which are used to characterize the methicillin-resistance phenotype, differed significantly between the MRSA and MSSA groups (both FDR-adjusted *p* < 0.001).

### 3.3. Prevalence of Species-Confirmatory, Resistance, and Virulence Genotypes

The overall prevalence of the molecular markers among the 100 *S. aureus* isolates is presented in Table 4. Real-Time PCR confirmed the presence of the species-specific *nuc* gene in 100.0% (*n* = 100) of the isolates. Regarding resistance determinants, 55.0% (*n* = 55) of the strains carried *mecA*. Carriage of *pvl* was identified in 25.0% (*n* = 25) of the total population. Among the virulence regulators, *agr I* was the predominant allele (35.0%), followed by *agr III* (22.0%) and *agr II* (12.0%). *Agr IV* was found in only a single isolate (1.0%).

### 3.4. Phenotypic-Genotypic Concordance and Association of agr Groups with Methicillin Resistance

In accordance with standard EUCAST interpretive guidelines, phenotypic methicillin susceptibility profiles (oxacillin susceptibility and cefoxitin screening) demonstrated complete (100%) concordance with molecular *mecA* PCR detection across all evaluated isolates.

Methicillin-resistance status was significantly associated with patient setting (*p* = 0.044). MRSA isolates were recovered more frequently from outpatients than MSSA isolates (56.4% vs. 37.8%), whereas MSSA isolates were more frequently recovered from inpatient wards (55.6% vs. 30.9%).

After Benjamini–Hochberg false discovery rate correction, *pvl* positivity remained significantly more frequent among MRSA isolates than among MSSA isolates (40.0% vs. 6.7%; FDR-adjusted *p* < 0.001). Overall, *agr* groups were successfully determined in 70.0% (*n* = 70) of the isolates, while the remaining 30.0% (*n* = 30; 18 MRSA (32.7%) and 12 MSSA (26.7%)) were classified as non-typeable (*agr*-negative). The distribution of typeable *agr* groups also differed according to methicillin-resistance status. *Agr I* positivity was less frequent among MRSA isolates than among MSSA isolates (21.8% vs. 51.1%; FDR-adjusted *p* = 0.004), whereas *agr II* positivity was more frequent among MRSA isolates (20.0% vs. 2.2%; FDR-adjusted *p* = 0.010). No significant difference was observed for *agr III* (25.5% vs. 17.8%; FDR-adjusted *p* = 0.357). *Agr IV* was detected in only one MSSA isolate and in none of the MRSA isolates, and this difference was not statistically significant (Fisher’s exact *p* = 0.267; FDR-adjusted *p* = 0.320) (Table 5). All isolates were *nuc* positive confirming their identification as *S. aureus*; therefore, no between-group comparisons were performed for this species-specific marker.

**Table 5:** Distribution of molecular markers and *agr* polymorphic groups stratified by methicillin resistance status.

	Result	MRSA *n* (%)	MSSA *n* (%)	*p*-Value	FDR-Adjusted *p*
*nuc*	Positive	55 (100.0)	45 (100.0)	—	-
	Negative	0 (0.0)	0 (0.0)		
*mecA*	Positive	55 (100.0)	0 (0.0)	<0.001	<0.001
	Negative	0 (0.0)	45 (100.0)		
*pvl*	Positive	22 (40.0)	3 (6.7)	<0.001	<0.001
	Negative	33 (60.0)	42 (93.3)		
*agr I*	Positive	12 (21.8)	23 (51.1)	0.002	0.004
	Negative	43 (78.2)	22 (48.9)		
*agr II*	Positive	11 (20.0)	1 (2.2)	0.006	0.010
	Negative	44 (80.0)	44 (97.8)		
*agr III*	Positive	14 (25.5)	8 (17.8)	0.357	0.357
	Negative	41 (74.5)	37 (82.2)		
*agr IV*	Positive	0 (0.0)	1 (2.2)	0.267	0.320
	Negative	55 (100.0)	44 (97.8)		

### 3.5. Distribution of Molecular Markers, agr Groups, and Antimicrobial Resistance Profiles According to pvl Carriage

The distribution of *agr* groups and antimicrobial susceptibility profiles according to *pvl* carriage demonstrated statistically significant epidemiological patterns. When the distribution of *agr* alleles was evaluated based on *pvl* status (Table 6), a statistically significant difference was observed. After Benjamini–Hochberg false discovery rate correction, the distribution of selected molecular markers differed between *pvl*-positive and *pvl*-negative isolates. *agr II* positivity was more frequent among *pvl*-positive isolates than among *pvl*-negative isolates (40.0% vs. 2.7%; FDR-adjusted *p* < 0.001). In contrast, *agr I* positivity was less frequent among *pvl*-positive isolates (4.0% vs. 45.3%; FDR-adjusted *p* < 0.001). No significant difference was observed between the groups in the distribution of *agr III* (16.0% vs. 24.0%; FDR-adjusted *p* = 0.504). *Agr IV* was detected in only one *pvl*-negative isolate and in none of the *pvl*-positive isolates; this difference was not statistically significant (Fisher’s exact *p* > 0.999; FDR-adjusted *p* > 0.999). *MecA* positivity was more frequent among *pvl*-positive isolates than among *pvl*-negative isolates (88.0% vs. 44.0%; FDR-adjusted *p* < 0.001). All isolates were *nuc* positive; therefore, no between-group comparisons were performed for this species-specific marker.

When phenotypic antimicrobial resistance profiles were cross-analyzed with *pvl* status (Table 7), *pvl*-positive isolates demonstrated higher resistance frequencies to specific non-beta-lactam agents. After Benjamini–Hochberg false discovery rate correction, erythromycin resistance (72.0% vs. 28.0%; FDR-adjusted *p* < 0.001), fusidic acid resistance (56.0% vs. 20.0%; FDR-adjusted *p* = 0.001), and mupirocin resistance (44.0% vs. 8.0%; FDR-adjusted *p* < 0.001) were more frequent among *pvl*-positive isolates than among *pvl*-negative isolates. Concordantly, phenotypic markers for methicillin resistance, including cefoxitin-screen positivity (88.0% vs. 44.0%; FDR-adjusted *p* < 0.001) and oxacillin resistance (88.0% vs. 44.0%; FDR-adjusted *p* < 0.001) were also more frequent among *pvl*-positive isolates.

In contrast, constitutive clindamycin resistance was not detected among *pvl*-positive isolates but was present in 24.0% of *pvl*-negative isolates (0.0% vs. 24.0%; FDR-adjusted *p* = 0.010). Similarly, inducible clindamycin resistance was observed only among *pvl*-negative isolates (0.0% vs. 21.3%; FDR-adjusted *p* = 0.017). Levofloxacin resistance (0.0% vs. 17.3%; FDR-adjusted *p* = 0.046) and tetracycline resistance (0.0% vs. 18.7%; FDR-adjusted *p* = 0.028) were also restricted to the *pvl*-negative group. No significant differences were observed between the groups in resistance to penicillin, daptomycin, and TMP-SXT (all FDR-adjusted *p* > 0.05).

### 3.6. Factors Associated with Methicillin Resistance

To evaluate factors independently associated with methicillin resistance among clinical *S. aureus* isolates while controlling for potential demographic confounders, a multivariable Firth’s penalized logistic regression analysis was conducted on the cohort of 100 non-duplicate clinical isolates (Table 8). Potential confounding parameters, including demographic variables (age and sex) and *pvl* carriage, were evaluated in the multivariable model. Multivariable Firth’s penalized logistic regression model was employed to adjust for potential small-sample bias. After adjustment for age and sex, *pvl*-positive isolates had significantly higher odds of exhibiting the MRSA phenotype compared to *pvl*-negative isolates (adjusted *OR*: 8.32, 95% *CI*: 2.68–34.09; *p* < 0.001). Neither sex (adjusted OR: 0.74, 95% CI: 0.31–1.75; *p* = 0.488) nor age (adjusted OR: 1.07, 95% CI: 0.88–1.31; *p* = 0.506) was significantly associated with methicillin resistance status. The apparent AUC was 0.712, whereas the optimism-corrected AUC was 0.674, indicating modest model discrimination.

Model diagnostics confirmed robust specification: standardized Pearson residuals ranged from −2.664 to 1.400, and standardized deviance residuals ranged from −2.072 to 1.491. Three observations had absolute standardized Pearson residuals greater than 2, and two had absolute standardized deviance residuals greater than 2; however, no standardized residual exceeded an absolute threshold of 3. The maximum leverage value was 0.070, which was below the prespecified threshold of 0.080. Although three observations exceeded the Cook’s distance screening threshold of 0.040, the maximum Cook’s distance was 0.090, and no value exceeded 1. The case-deletion analysis showed that the direction and magnitude of the independent association between *pvl* carriage and the MRSA phenotype remained stable. Variance Inflation Factor (VIF) values ranged from 1.03 to 1.07, indicating no problematic multicollinearity. The Hosmer–Lemeshow test showed no evidence of lack of fit (*χ*^2^ = 4.88, *df* = 8; *p* = 0.771). The calibration intercept and slope were 0.01 and 1.08, respectively, and the Brier score was 0.2.

## 4. Discussion

*S. aureus* strains carry genes that confer resistance to various antibiotics, including anti-staphylococcal drugs. The chromosomal structure carrying the gene responsible for cefoxitin resistance may also contain genes and regulatory elements capable of conferring resistance to numerous antibiotics, such as clindamycin, aminoglycosides, fluoroquinolones, macrolides, co-trimoxazole and chloramphenicol. The high mortality rates associated with MRSA infections have led to these strains being characterised in detail in terms of their resistance profiles, molecular epidemiology and virulence factors. To the best of our knowledge, this laboratory-based study represents the first comprehensive investigation in our region to simultaneously conduct a thorough cross-analysis of the *mecA*, *pvl* and *agr* groups (I–IV), directly correlating them with comprehensive phenotypic in vitro antimicrobial susceptibility profiles. The most notable finding of this molecular surveillance is the strong statistical and epidemiological association identified between *pvl* carriage and methicillin resistance, with multivariable Firth’s penalized regression confirming that *pvl* carriage was independently associated with an over eight-fold increase in the odds of the MRSA phenotype (adjusted OR = 8.32, 95% CI: 2.68–34.09, *p* < 0.001). Rather than indicating a direct causal relationship, this finding reflects the concurrent circulation of *pvl*-positive MRSA isolates within our center. Given the modest sample size (*n*=100) and the relatively wide confidence intervals, these results represent exploratory epidemiological associations that warrant validation in larger, multicenter genomic studies. At the same time, our cross-analytical approach revealed a distinct genotypic distribution pattern: whilst the *agr* group II was more frequently detected within the *pvl*-positive population, *agr* group I was significantly predominant among MSSA isolates. Most importantly, the fact that resistance to clindamycin, levofloxacin, and tetracycline was entirely confined to the *pvl*-negative cohort demonstrates a distinct antimicrobial susceptibility profile in this study population, where *pvl*-positive isolates frequently maintained susceptibility to these non-beta-lactam agents despite harboring methicillin resistance determinants.

The epidemiological distribution of *S. aureus* strains reflects regional antimicrobial usage and resistance patterns. In this study, a high MRSA prevalence (55.0%) was identified, which aligns closely with data from the Eastern Mediterranean region (e.g., 57.45% in Crete, Greece by Vittorakis et al. [1]) but contrasts sharply with lower rates reported across Northern and Western Europe [1]. Crucially, these epidemiological discrepancies are shaped not only by geographical differences but also by distinct antimicrobial stewardship policies, infection control infrastructure, and hospitalization dynamics.

Furthermore, while national multicenter surveillance in Turkey by Cikman et al. [20] predominantly captured patients with prolonged hospitalizations (reporting 47.9% of MRSA exclusively from ICUs). Similarly, Sağlam et al. (2024) found that sefoxitin-resistant MRSA is more prevalent among inpatients [21]. Our cohort was distinguished by a high proportion of outpatient isolates (56.4%), whilst MSSA isolates were found to be more prevalent in inpatient wards (55.6%; *p* = 0.044). This clinical distribution differs from traditional paradigms in which methicillin resistance is closely associated with ICUs and prolonged hospital stays [20,21]. This methodological and demographic difference in sampling frames reflects a substantial recovery of MRSA isolates from outpatient clinical settings rather than an isolation pattern restricted exclusively to ICUs or inpatient wards within our hospital; however, definitive delineation of community transmission dynamics would require systematic epidemiological contact tracing and molecular lineage typing.

In terms of sample types, wound infections (52.7% vs. 51.1%) and blood cultures (27.3% vs. 24.4%) emerged as the most common sources in both cohorts. This trend contrasts with some of the more extensive genetic studies conducted in Turkey, such as those by Cikman et al. (2019) and Sağlam et al. (2024), in which respiratory samples predominate [20,21]. This clinical distinction is critical: respiratory isolates frequently harbor multidrug-resistant nosocomial clones, whereas purulent skin and soft tissue infections represent common clinical sources for toxin-carrying isolates. In contrast, our sample shows a strong correlation with the study conducted by Ceylan et al. (2022) in Istanbul; in that study, skin, soft tissue and blood cultures formed the basis of the clinical MRSA isolates [15]. A similar distribution pattern has also been documented in Greece by Vittorakis et al. (2023) [1]; in this study, abscesses, wound ulcers and blood samples constituted the primary clinical matrices. The marked predominance of purulent skin lesions in our study is consistent with the frequent detection of *pvl* and the distinct non-beta-lactam susceptibility patterns observed.

A cross-analysis of in vitro susceptibility profiles revealed that penicillin resistance was absolute (100.0%) in MRSA isolates and significantly higher than in the MSSA group (75.6%; FDR-adjusted *p* < 0.001). This comprehensive resistance profile is consistent with the long-standing nationwide trends in beta-lactamase production; this was also demonstrated in a pediatric surveillance study previously conducted by Karbuz et al. (2017), which documented a penicillin resistance rate of 93.8% [22]. Furthermore, our MRSA isolates exhibited statistically significantly higher resistance rates for erythromycin (61.8% vs. 11.1%; FDR-adjusted *p* < 0.001) and fusidic acid (41.8% vs. 13.3%; FDR-adjusted *p* = 0.004) compared with the MSSA group. The distribution of this non-beta-lactam multidrug resistance varies significantly across different geographical regions and centres in Turkey. For instance, while Karbuz et al. [22] documented remarkably low erythromycin (6.9%) and clindamycin (5.0%) resistance rates in Ankara, that investigation was strictly restricted to a pediatric patient population where antibiotic selection pressure and cumulative lifetime exposure are inherently lower than in our adult-dominated tertiary care cohort. Conversely, our observed resistance profiles parallel inpatient cohorts (e.g., Ceylan et al. [15] in Istanbul; 79.0% erythromycin resistance and 34.0% clindamycin resistance), which is consistent with local patterns of empirical macrolide and lincosamide utilization in clinical practice.

Our analysis showed that clindamycin resistance was numerically more frequent in the MRSA cohort than in the MSSA population (25.5% vs. 8.9%; *p* = 0.032), although this difference did not retain statistical significance after multiple-testing correction (FDR-adjusted *p* = 0.064). Similarly, inducible clindamycin resistance (iMLSb; 21.8% vs. 8.9%; FDR-adjusted *p* = 0.119) and mupirocin resistance (23.6% vs. 8.9%; FDR-adjusted *p* = 0.087) were numerically higher among MRSA isolates but did not reach statistical significance after FDR adjustment. In contrast, earlier data published by Chan et al. (2024) in Malaysia reported that resistance to clindamycin and erythromycin was statistically significantly higher, particularly in MRSA isolates (*p* < 0.05); Derakhshan et al. (2021), on the other hand, highlighted an inverse relationship, noting that antibiotic susceptibility was generally associated with a robust biofilm and was higher in *agr*-positive genotypes [13,14]. Reassuringly, all strains assessed in both cohorts demonstrated absolute susceptibility (100.0%) to vancomycin, teicoplanin, linezolid, tigecycline and nitrofurantoin. This preservation of reserve therapeutics strongly supports current studies in our country, as evidenced by the findings of Sağlam et al. (2024) and Ceylan et al. (2022), and is consistent with the global indicators presented by Chan et al. (2024). According to these indicators, no variants resistant to linezolid and vancomycin have been detected, indicating that these essential agents retain their full clinical efficacy in our tertiary care facility [14,15,21].

The molecular analysis of methicillin resistance determinants in our cohort demonstrated that 55.0% of the clinical isolates harbored the *mecA* gene; this finding showed complete (100.0%) agreement with phenotypic cefoxitin screening and oxacillin susceptibility profiles (Cohen’s kappa coefficient = 1.000). This complete agreement between phenotypic cefoxitin resistance and *mecA* carriage corroborates broader surveillance data in Turkey, including national studies by Ceylan et al. (2022) and Cikman et al. (2019) confirming that standard laboratory disk diffusion and automated screening maintain high diagnostic reliability for detecting classical *mecA*-mediated resistance in our region [15,20].

At the same time, a cross-analysis of *agr* groups stratified by methicillin resistance status (Table 5) revealed striking and statistically significant patterns of genotypic distribution within our clinical *S. aureus* cohort. The prevalence of *agr I* in the MSSA cohort was statistically significantly higher than in the MRSA group (51.1% vs. 21.8%; FDR-adjusted *p* = 0.004); this finding largely aligns with the baseline pediatric data obtained by Karbuz et al. (2017) in Ankara, where *agr I* constituted the predominant background of susceptible strains [22]. In contrast, the *agr* group *II* showed a highly significant enrichment in the MRSA cohort compared to the MSSA group (20.0% vs. 2.2%; FDR-adjusted *p* = 0.010); this finding is consistent with recent global data published by Chan et al. (2024), who noted correlations between *agr* locus polymorphism and resistance to oxacillin and cefoxitin in Malaysia [14]. Although no significant difference was observed for *agr III* (17.8% for MSSA vs 25.5% for MRSA), the rarity of *agr IV* (1.0% overall; limited to a single MSSA strain) is in excellent agreement with the multicenter profiles reported by Vittorakis et al. (2023) and Derakhshan et al. (2021), confirming that *agr* groups I and III represent the predominant circulating genotypes among typeable isolates [1,13]. Beyond the typeable groups, a proportion of our isolates (30.0%, *n* = 30; 18 MRSA and 12 MSSA) were categorized as non-typeable, a finding consistent with previously published clinical series where non-typeability typically ranges between 10% and 35%. This non-typeable profile can arise from nucleotide polymorphisms or point mutations at primer-binding sites within the hypervariable *agrB-D-C* region, novel divergent alleles, or partial deletions within the *agr* operon associated with *agr* dysfunction [23].

A contingency table analysis of the relationship between *pvl* carriage, *agr* groups, and resistance profiles (Table 6) demonstrated statistically significant epidemiological associations. According to these patterns isolates carrying the *pvl* marker demonstrated a higher frequency of *agr* group II (40.0% vs. 2.7%; FDR-adjusted *p* < 0.001). While this specific distribution reflects a regional epidemiological pattern, the strong association between certain *agr* genotypes and resistance determinants have also been reported by Karbuz et al. (2017) and Chan et al. (2024) [14,22]. It should be emphasized that our genotyping identifies structural polymorphism within the *agr* locus rather than quantitative gene expression (such as RNAIII transcript levels) or functional regulatory activity; thus, these findings represent molecular epidemiological distribution patterns rather than direct variations in pathogenic potential in vivo. Furthermore, our genotypic data revealed that *mecA* carriage was statistically significantly more prevalent among *pvl* carriers than among non-*pvl* carriers (88.0% vs. 44.0%; FDR-adjusted *p* < 0.001); this finding strongly reflects the molecular profile reported by Sağlam et al. (2024) [21]. In that study, *pvl* determinants were shown to be significantly more prevalent among MRSA isolates than MSSA isolates, demonstrating a strong epidemiological association.

When phenotypic antimicrobial resistance profiles were cross-analyzed with virulence status (Table 7), *pvl*-positive strains exhibited significantly higher resistance rates than *pvl*-negative isolates for erythromycin (72.0% vs. 28.0% FDR-adjusted *p* < 0.001), fusidic acid (56.0% vs. 20.0% FDR-adjusted *p* = 0.001), and mupirocin (44.0% vs. 8.0% FDR-adjusted *p* ≤ 0.001). This pattern of multidrug resistance is consistent with global observations reported by Karmakar et al. (2018); these researchers noted that *pvl*-positive clinical isolates frequently present concurrent non-beta-lactam resistance profiles [12]. In contrast, the fact that constitutive and inducible clindamycin resistance were confined entirely to our *pvl*-negative group (24.0%, FDR-adjusted *p* = 0.010 and 21.3%, FDR-adjusted *p* = 0.017, respectively; compared to 0.0% in *pvl*-positive isolates) is consistent with the marked trends in resistance differentiation observed in local surveillance studies. This trend is consistent with the findings reported by Karbuz et al. (2017) and Chan et al. (2024); these studies demonstrated that *pvl*-positive isolates are typically susceptible to clindamycin, whereas clindamycin resistance is predominantly observed in *pvl*-negative strains [14,22]. Our findings indicate that resistance to clindamycin, levofloxacin, and tetracycline is limited to *pvl*-negative isolates. This observation indicates that *pvl*-positive isolates in this study population exhibited preserved susceptibility to clindamycin and fluoroquinolones, while broader multidrug resistance was confined to *pvl*-negative strains within this cohort.

To further assess the factors independently associated with methicillin resistance while accounting for potential demographic confounders and minimizing the risk of model overfitting, a multivariable Firth’s penalized logistic regression analysis was conducted (Table 8). In this adjusted model, *pvl* carriage remained strongly and independently associated with the methicillin resistance, with *pvl*-positive isolates exhibiting an over eight-fold higher odds of being MRSA compared to *pvl*-negative isolates (OR = 8.32, 95% CI: 2.68–34.09, *p* < 0.001). In contrast, demographic variables including age (adjusted OR = 1.07, 95% CI: 0.88–1.31, *p* = 0.506) and male sex (adjusted OR = 0.74, 95% CI: 0.31–1.75, *p* = 0.488) were not significantly associated with methicillin resistance status. Rather than implying a direct causal relationship, this statistical association highlights an epidemiological association and demonstrates that *pvl*-positive isolates frequently co-circulate with the MRSA phenotype in our clinical setting. Furthermore, this strong association highlights a notable epidemiological overlap of key virulence and structural resistance determinants in local outpatient settings; the clinical challenge posed by this concurrent carriage has previously been emphasized by Karbuz et al. (2017) and Sağlam et al. (2024) as a significant concern for regional empirical treatment protocols [21,22].

Although this study provides important epidemiological insights into the concurrent carriage and distribution of resistance and virulence determinants, it is essential to acknowledge certain limitations. Primarily, this study employs a single-center cross-sectional design that reflects specific patient dynamics at a tertiary care hospital rather than a general nationwide distribution. Regional data show that antibiotic resistance patterns are shaped by local dynamics; this underscores that multicenter surveillance studies are essential for accurately tracking long-term epidemiological trends and the distribution of resistant clones across the country. In addition, although our real-time PCR system has successfully demonstrated the perfect correlation between genotype and phenotype, this target-specific method is limited to our predefined primer panel; this means that we cannot screen for alternative concurrent resistance mutations or new virulence factors outside of these specific target genes. Furthermore, while SYBR Green real-time PCR provided rapid qualitative screening, the primer pairs were adapted from conventional PCR protocols, generating longer amplicons (279–588 bp) than typically recommended for qPCR assays. Although assay specificity was strictly controlled through melting curve dissociation criteria and standard reference strains (*S. aureus* ATCC 43300 and ATCC 25923) for methicillin resistance verification, verified positive reference strains were not available in our laboratory for *pvl* and *agr* groups (I–IV). Additionally, because real-time PCR was utilized strictly for qualitative presence/absence screening from pure bacterial isolates, formal analytical validation metrics for quantitative assays (such as standard curves and limits of detection) were not determined. Moreover, molecular evaluation across all isolates was strictly limited to qualitative genotypic identification without functional gene expression analyses or phenotypic exoprotein assays. Furthermore, high-resolution molecular typing methods, such as multilocus sequence typing (MLST), *spa* typing, SCC*mec* characterization, or whole-genome sequencing (WGS), were not performed on any of the isolates. Consequently, our findings reflect local epidemiological associations and concurrent gene carriage patterns rather than longitudinal transmission dynamics; future studies incorporating sequence-based typing are warranted to evaluate the molecular epidemiology of these clinical isolates. Finally, since this study was designed as a purely laboratory-based molecular characterization, it naturally lacked the ability to include comprehensive long-term clinical follow-up data or to monitor long-term patient outcomes following treatment.

## 5. Conclusions

In conclusion, this laboratory-based molecular study provides valuable institutional baseline data on the epidemiological co-occurrence of *pvl* carriage and specific antimicrobial resistance profiles within our tertiary care center. Our real-time PCR platform demonstrated complete concordance between *mecA* detection and phenotypic cefoxitin resistance, reinforcing the reliability of rapid molecular screening in hospital diagnostic workflows. Most importantly, multivariable Firth’s penalized logistic regression identified *pvl* carriage as independently associated with the MRSA phenotype in our clinical cohort. While the distribution of *pvl*-positive isolates within specific *agr* groups and the concurrent presence of non-betalactam resistance phenotypes highlight important local surveillance patterns, these observations represent single-center findings derived from targeted PCR screening. Accordingly, whilst these data contribute to local antimicrobial stewardship practices and raise awareness of infection control at an organisational level, there is a need for more comprehensive multicentre studies that combine genomic sequencing with longitudinal clinical outcome data in order to develop generalisable clinical guidelines and determine more comprehensive empirical treatment strategies.

## Figures and Tables

**Table 2:** Demographic and Clinical Characteristics of Patients with MRSA and MSSA Isolates.

Characteristic	MRSA (*n* = 55)	MSSA (*n* = 45)	*p*-Value
Age, years	Median (Q1, Q3)	Median (Q1, Q3)	
	38 (14, 61)	41 (25, 58)	0.623
Sex			
Female, *n* (%)	26 (47.3)	17 (37.8)	0.340
Male, *n* (%)	29 (52.7)	28 (62.2)	
Patient setting			
Outpatient, *n* (%)	31 (56.4)	17 (37.8)	0.044
Inpatient, *n* (%)	17 (30.9)	25 (55.6)	
Intensive Care Unit (ICU), *n* (%)	7 (12.7)	3 (6.7)	
Specimen type			
Blood culture, *n* (%)	15 (27.3)	11 (24.4)	0.647
Wound specimen, *n* (%)	29 (52.7)	23 (51.1)	
Urine, *n* (%)	1 (1.8)	3 (6.7)	
Respiratory samples, *n* (%) (endotracheal aspirates, bronchoalveolar lavage, and sputum)	9 (16.4)	6 (13.3)	
Sterile body fluid, *n* (%) (pleural fluid, cerebrospinal fluid)	1 (1.8)	1 (2.2)	
Conjunctival swabs *n* (%)	0 (0.0)	1 (2.2)	

**Table 3:** Phenotypic antimicrobial susceptibility profiles of the *S. aureus* isolates stratified by methicillin resistance status.

Antimicrobial Agent	Result	MRSA *n* (%)	MSSA *n* (%)	*p*-Value	FDR-Adjusted *p*
Cefoxitin screen	Positive	55 (100.0)	0 (0.0)	<0.001	<0.001
	Negative	0 (0.0)	45 (100.0)		
Inducible clindamycin resistance	Positive	12 (21.8)	4 (8.9)	0.079	0.119
	Negative	43 (78.2)	41 (91.1)		
Clindamycin	S	41 (74.5)	41 (91.1)	0.032	0.064
	R	14 (25.5)	4 (8.9)		
Daptomycin	S	54 (98.2)	45 (100.0)	>0.999	>0.999
	R	1 (1.8)	0 (0.0)		
Erythromycin	S	21 (38.2)	40 (88.9)	<0.001	<0.001
	R	34 (61.8)	5 (11.1)		
Fusidic acid	S	32 (58.2)	39 (86.7)	0.002	0.004
	R	23 (41.8)	6 (13.3)		
Levofloxacin	S	47 (85.5)	40 (88.9)	0.611	0.734
	R	8 (14.5)	5 (11.1)		
Linezolid	S	55 (100.0)	45 (100.0)	—	-
	R	0 (0.0)	0 (0.0)		
Mupirocin	S	42 (76.4)	41 (91.1)	0.051	0.087
	R	13 (23.6)	4 (8.9)		
Nitrofurantoin	S	55 (100.0)	45 (100.0)	—	-
	R	0 (0.0)	0 (0.0)		
Oxacillin	S	0 (0.0)	45 (100.0)	<0.001	<0.001
	R	55 (100.0)	0 (0.0)		
Penicillin	S	0 (0.0)	11 (24.4)	<0.001	<0.001
	R	55 (100.0)	34 (75.6)		
Teicoplanin	S	55 (100.0)	45 (100.0)	—	-
	R	0 (0.0)	0 (0.0)		
Tetracycline	S	45 (81.8)	41 (91.1)	0.183	0.244
	R	10 (18.2)	4 (8.9)		
Tigecycline	S	55 (100.0)	45 (100.0)	—	-
	R	0 (0.0)	0 (0.0)		
TMP-SXT	S	51 (92.7)	43 (95.6)	0.688	0.750
	R	4 (7.3)	2 (4.4)		
Vancomycin	S	55 (100.0)	45 (100.0)	—	-
	R	0 (0.0)	0 (0.0)		

**Table 4:** Distribution of species-confirmatory, antimicrobial resistance and virulence genes among clinical *S. aureus* isolates (*n* = 100).

	Gene Category	Positive *n* (%)	Negative *n* (%)
*nuc*	Species confirmation	100 (100.0)	0 (0.0)
*mecA*	Antimicrobial resistance	55 (55.0)	45 (45.0)
*pvl*	Virulence factor	25 (25.0)	75 (75.0)
*agr I*	Virulence regulator	35 (35.0)	65 (65.0)
*agr II*	Virulence regulator	12 (12.0)	88 (88.0)
*agr III*	Virulence regulator	22 (22.0)	78 (78.0)
*agr IV*	Virulence regulator	1 (1.0)	99 (99.0)

**Table 6:** Association of *agr* groups with *pvl* carriage.

	Result	*pvl*-Positive *n* (%)	*pvl*-Negative *n* (%)	*p*-Value	FDR-Adjusted *p*
*nuc*	Positive	25 (100.0)	75 (100.0)	—	-
	Negative	0 (0.0)	0 (0.0)		
*mecA*	Positive	22 (88.0)	33 (44.0)	<0.001	<0.001
	Negative	3 (12.0)	42 (56.0)		
*agr I*	Positive	1 (4.0)	34 (45.3)	<0.001	<0.001
	Negative	24 (96.0)	41 (54.7)		
*agr II*	Positive	10 (40.0)	2 (2.7)	<0.001	<0.001
	Negative	15 (60.0)	73 (97.3)		
*agr III*	Positive	4 (16.0)	18 (24.0)	0.403	0.504
	Negative	21 (84.0)	57 (76.0)		
*agr IV*	Positive	0 (0.0)	1 (1.3)	>0.999	>0.999
	Negative	25 (100.0)	74 (98.7)		

**Table 7:** Comparison of Antimicrobial Resistance Profiles according to *pvl*.

Antimicrobial Agent	Result	*pvl*-Positive *n* (%)	*pvl*-Negative *n* (%)	*p*-Value	FDR-Adjusted *p*
Cefoxitin screen	Positive	22 (88.0)	33 (44.0)	<0.001	<0.001
	Negative	3 (12.0)	42 (56.0)		
Inducible clindamycin resistance	Positive	0 (0.0)	16 (21.3)	0.010	0.017
	Negative	25 (100.0)	59 (78.7)		
Clindamycin	S	25 (100.0)	57 (76.0)	0.005	0.010
	R	0 (0.0)	18 (24.0)		
Daptomycin	S	25 (100.0)	74 (98.7)	>0.999	>0.999
	R	0 (0.0)	1 (1.3)		
Erythromycin	S	7 (28.0)	54 (72.0)	<0.001	<0.001
	R	18 (72.0)	21 (28.0)		
Fusidic acid	S	11 (44.0)	60 (80.0)	0.001	0.001
	R	14 (56.0)	15 (20.0)		
Levofloxacin	S	25 (100.0)	62 (82.7)	0.034	0.046
	R	0 (0.0)	13 (17.3)		
Linezolid	S	25 (100.0)	75 (100.0)	—	-
	R	0 (0.0)	0 (0.0)		
Mupirocin	S	14 (56.0)	69 (92.0)	<0.001	<0.001
	R	11 (44.0)	6 (8.0)		
Nitrofurantoin	S	25 (100.0)	75 (100.0)	—	-
	R	0 (0.0)	0 (0.0)		
Oxacillin	S	3 (12.0)	42 (56.0)	<0.001	<0.001
	R	22 (88.0)	33 (44.0)		
Penicillin	S	2 (8.0)	9 (12.0)	0.726	0.871
	R	23 (92.0)	66 (88.0)		
Teicoplanin	S	25 (100.0)	75 (100.0)	—	-
	R	0 (0.0)	0 (0.0)		
Tetracycline	S	25 (100.0)	61 (81.3)	0.019	0.028
	R	0 (0.0)	14 (18.7)		
Tigecycline	S	25 (100.0)	75 (100.0)	—	
	R	0 (0.0)	0 (0.0)		
TMP-SXT	S	24 (96.0)	70 (93.3)	>0.999	>0.999
	R	1 (4.0)	5 (6.7)		
Vancomycin	S	25 (100.0)	75 (100.0)	—	-
	R	0 (0.0)	0 (0.0)		

**Table 8:** Multivariable Firth’s penalized logistic regression analysis of factors independently associated with methicillin resistance in clinical *S. aureus* isolates (*n* = 100).

Variable	Final Model Adjusted OR (95% CI)	*p*-Value
*pvl* Carriage (Positive)	8.32 (2.68–34.09)	<0.001
Sex (Male)	0.74 (0.31–1.75)	0.488
Age	1.07 (0.88–1.31)	0.506

OR: Odds Ratio, CI: Confidence Interval. The multivariable model was prespecified and evaluated on the complete analytical cohort of 100 non-duplicate patient isolates using Firth’s penalized likelihood to minimize small-sample bias.

## Data Availability

The original contributions presented in this study are included in the article/Supplementary Material. Further inquiries can be directed to the corresponding author.

## Supplementary Materials

The following supporting information can be downloaded at: https://www.mdpi.com/article/10.3390/pathogens15090957/s1, Figure S1: Derivative melting peak profiles (-*dF*/*dT*) of the *nuc* (Tm = 80.5 ± 0.3 °C) confirming target-specific identification of clinical *S. aureus* isolates without non-specific products; Figure S2: Derivative melting peak profiles (-*dF*/*dT*) of the *mecA* (Tm = 79.5 ± 0.3 °C) in MRSA isolates; non-template controls and MSSA strains show flat baseline profiles; Figure S3: Derivative melting peak profiles (-*dF*/*dT*) of the *pvl* (Tm = 80.2± 0.3 °C ) in *pvl* positive isolates; negative isolates remain at baseline; Figure S4: Derivative melting peak profiles (-*dF*/*dT*) of *agr* group I isolates with a specific melting peak at Tm = 79.5 ± 0.3 °C; Figure S5: Derivative melting peak profiles (-*dF*/*dT*) of *agr* group II isolates with a specific melting peak at Tm = 77.5 ± 0.3 °C; Figure S6: Derivative melting peak profiles (-*dF*/*dT*) of *agr* group III isolates with a specific melting peak at Tm = 80.0 ± 0.3 °C; Figure S7: Derivative melting peak profile (-*dF*/*dT*) of the *agr* group IV-positive isolate displaying a discrete peak at Tm = 79.8 °C.
