# Supplementary material for: Molecular Characterization of Antimicrobial Resistance and Virulence Genotypes in Staphylococcus aureus Isolates: Investigation of mecA, pvl, and agr Groups
Source: Pathogens. 2026 Sep 8;15(9):957. doi: 10.3390/pathogens15090957 (PMC13610699; doi:10.3390/pathogens15090957)
Supplement: Supplementary file 1 [file pathogens-15-00957-s001.zip › pathogens-4471344-supplementary.pdf]

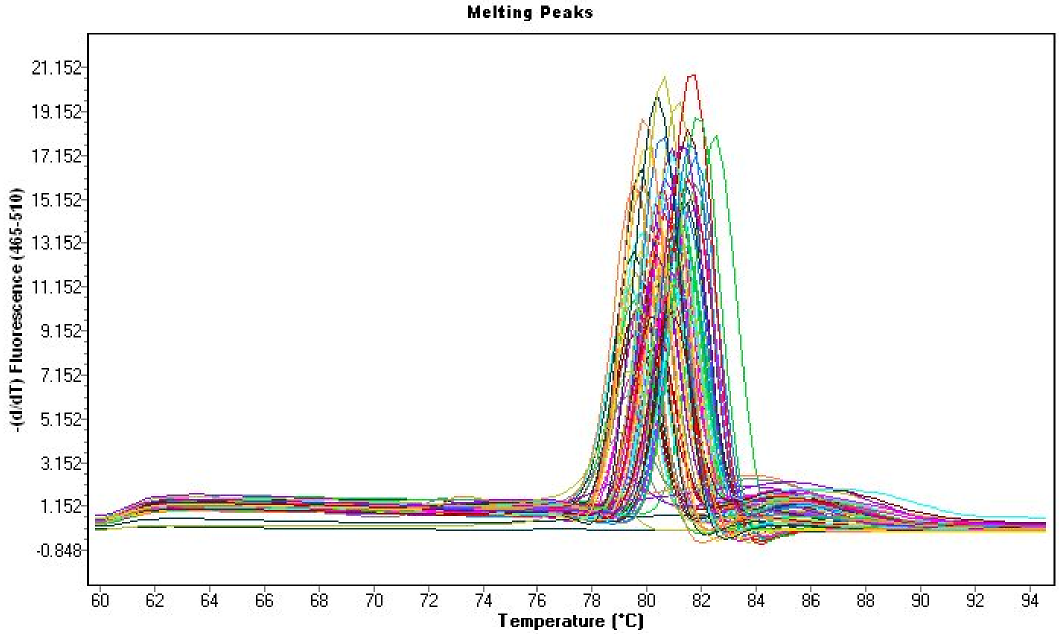

Figure S1. Derivative melting peak profiles (*-dF/dT*) of the *nuc* (T_m = 80.5 ± 0.3°C) confirming target-specific identification of clinical *S. aureus* isolates without non-specific products

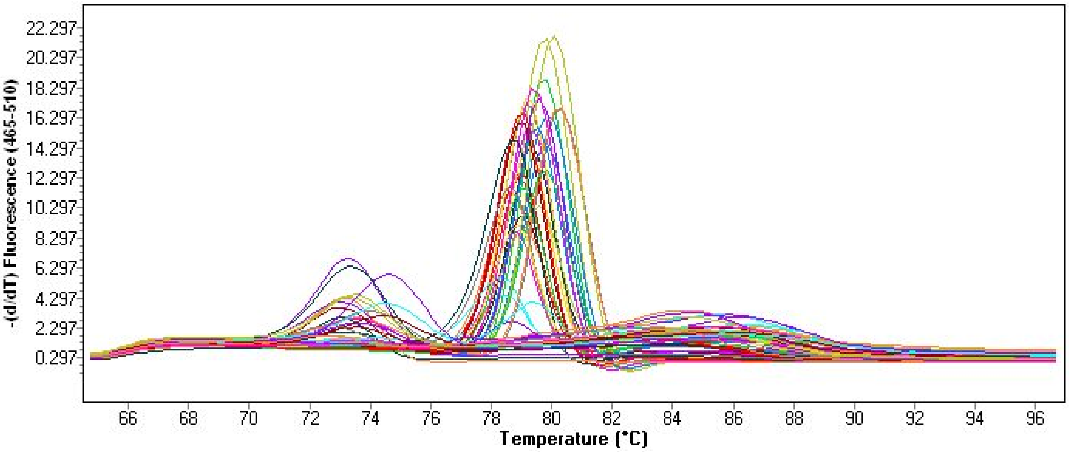

Figure S2. Derivative melting peak profiles (*-dF/dT*) of the *mecA* (T_m = 79.5 ± 0.3°C) in MRSA isolates; non-template controls and MSSA strains show flat baseline profiles

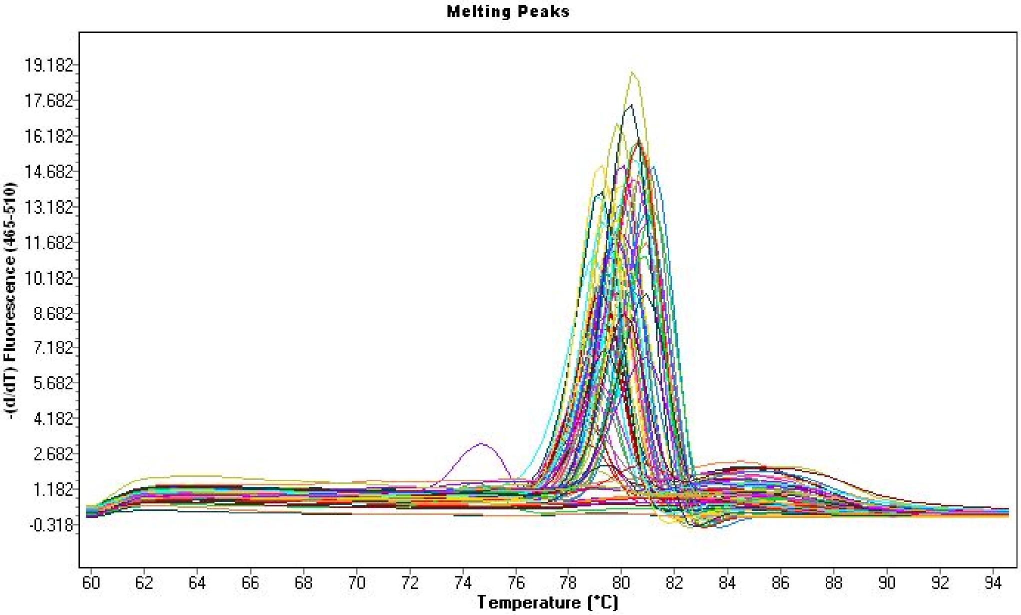

Figure S3. Derivative melting peak profiles *(-dF/dT)* of the *pvl* (T_m = 80.2± 0.3°C) in *pvl*-positive isolates; negative isolates remain at baseline

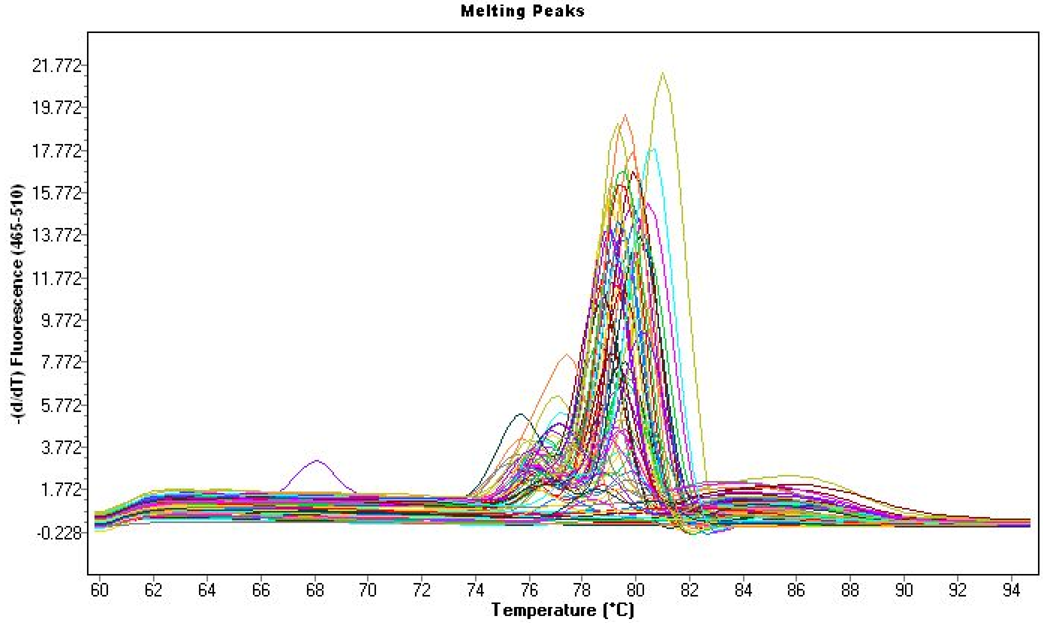

Figure S4. Derivative melting peak profiles (*-dF/dT*) of *agr* group I isolates with a specific melting peak at T_m = 79.5 ± 0.3°C

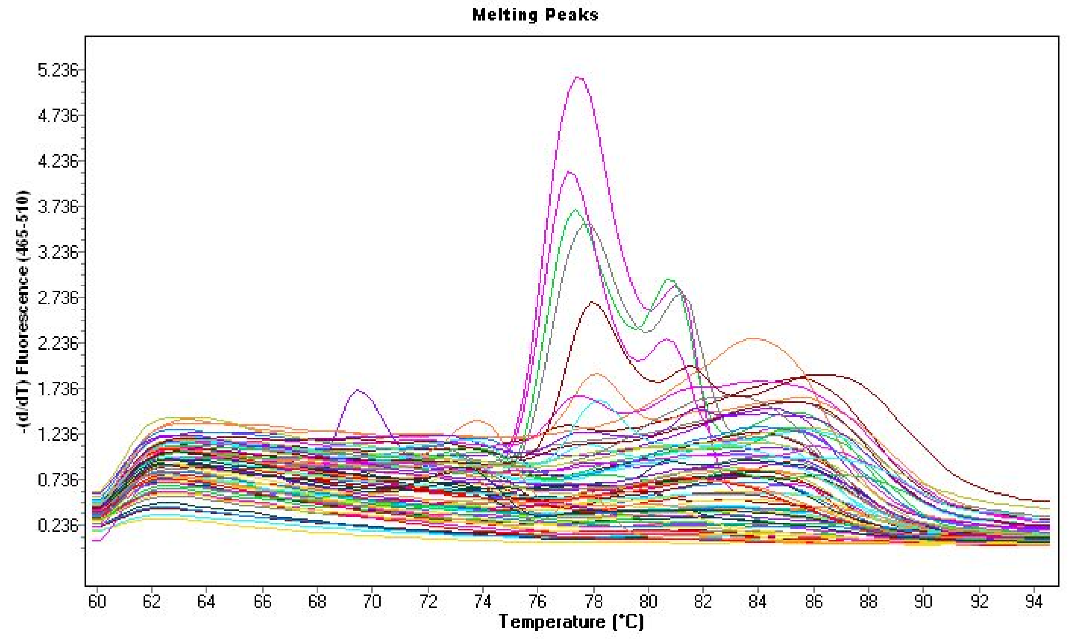

Figure S5. Derivative melting peak profiles (-*dF*/*dT*) of *agr* group II isolates with a specific melting peak at T_m = 77.5 ± 0.3°C

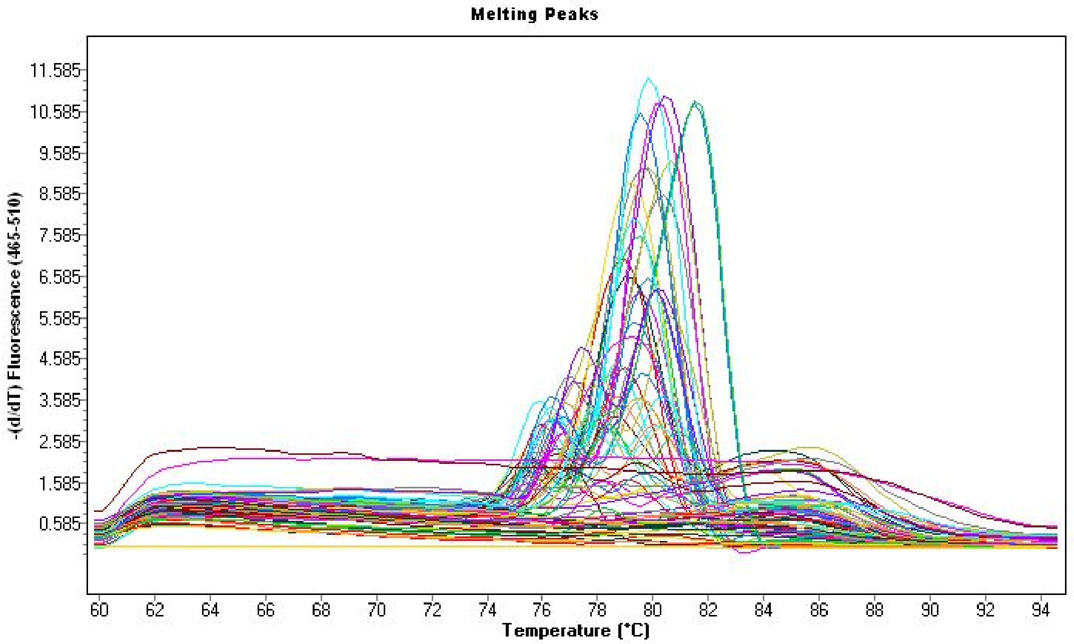

Figure S6. Derivative melting peak profiles (-dF/dT) of *agr* group III isolates with a specific melting peak at T_m = 80.0 ± 0.3°C

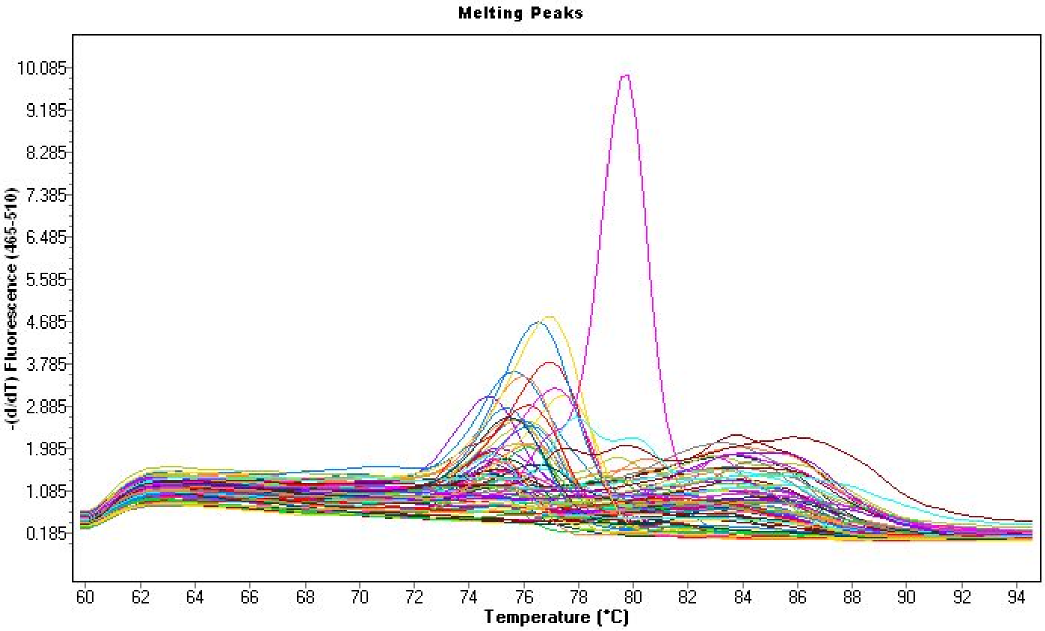

Figure S7. Derivative melting peak profile (-*dF*/*dT*) of the *agr* group IV-positive isolate displaying a discrete peak at T_m = 79.8°C
